# Supplementary material for: A Novel Gold Calreticulin Nanocomposite Based on Chitosan for Wound Healing in a Diabetic Mice Model
Source: Nanomaterials (Basel). 2019 Jan 8;9(1):75. doi: 10.3390/nano9010075 (PMC6359502; doi:10.3390/nano9010075)
Supplement: Supplementary file 1 [file nanomaterials-09-00075-s001.pdf]

nanomaterials

MDPI

Supplementary Materials

Table S1. Comparations of body weight and blood glucose in healthy and diabetic mice before and after treatments.

Groups	Body Weight (g) Before treatment	Body Weight (g) After treatment	Blood glucose (mg/dL) Before treatment	Blood glucose (mg/dL) After treatment
Healthy (buffer citrate)	23.73±1.5	24.30±0.66	90±7.48	100.32±5.06
Diabetic (SZT)	24.397±0.319	25.44±0.94**	92±8.03	221.46±7.51***

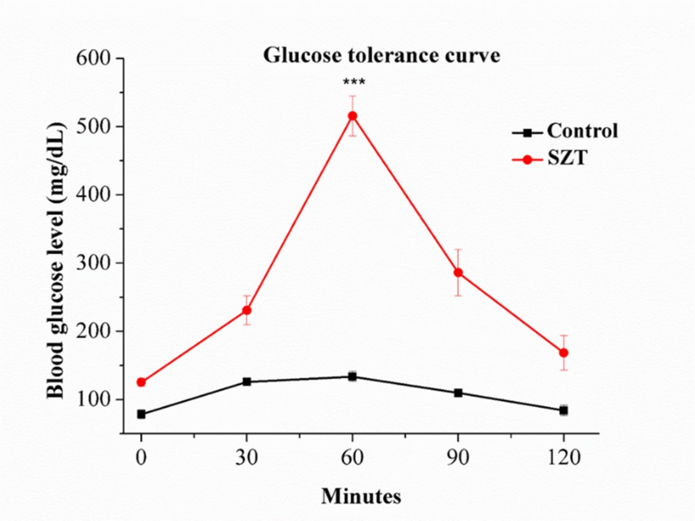

Figure S1. Glucose tolerance test. Concentration of blood glucose in healthy and diabetic mice after received dextrose orally. Significance difference was observed at 60 minutes (*** $p<0.0001$).
